# Remembering the Dying Days: Older Adults’ Final Memories From the Loss of a Spouse

**DOI:** 10.1093/geroni/igaa057.2048

**Published:** 2020-12-16

**Authors:** Emily Mroz, Susan Bluck

**Affiliations:** University of Florida, Gainesville, Florida, United States

## Abstract

Memories from the very end of the life of a deceased spouse (i.e., their dying days) are frequently carried with the bereaved as major markers in their own life stories. The current study identifies functions of these memories. Older adults (age 70-96; N = 53) told two memories from their spouse’s dying days, then self-rated them for serving directive, social-bonding and self-continuity functions (TALE; Bluck & Alea, 2011). Those who found their loss more incomprehensible (ISLES; Holland, 2015) reported using these memories for directive (i.e., guidance of behaviors) and self-continuity (i.e., maintenance of a sense of self) functions more frequently (ps < 0.05). This relation was, however, mediated by older adults’ current grief (ICG; Prigerson et al., 1995). Incomprehensibility of the loss of a spouse appears to lead to intense grieving, prompting individuals to draw on memories from the loss to maintain a sense of self and direct their future.

